# Inhibition of Abdominal Aortic Aneurysm Progression Through the CXCL12/CXCR4 Axis via MiR206‐3p Sponge

**DOI:** 10.1111/jcmm.70328

**Published:** 2025-01-08

**Authors:** Xuezhen Xuan, Yaling Li, Genmao Cao, Jie Hu, Sheng Yan, Haijiang Jin, Maolin Qiao, Ruijing Zhang, Honglin Dong

**Affiliations:** ^1^ Department of Vascular Surgery The Second Hospital of Shanxi Medical University Taiyuan China; ^2^ Department of Nephrology The Second Hospital of Shanxi Medical University Taiyuan China

**Keywords:** abdominal aortic aneurysm, CXCR4, miR206‐3p, phenotype transformation, vascular smooth muscle cells

## Abstract

Notably, the C‐X‐C Motif Chemokine Ligand 12/C‐X‐C Chemokine Receptor Type 4 (CXCL12/CXCR4) signalling pathway's activation is markedly increased in a mouse model of abdominal aortic aneurysms (AAA). Nonetheless, the precise contribution of this pathway to AAA development remains to be elucidated. The AAA mouse model was induced by local incubation with elastase and oral administration of β‐aminopropionitrile. The activity level of the CXCL12/CXCR4 axis was evaluated in both human AAA patients and the mouse model. Smooth muscle cell lineage tracing determined the expression and localisation of CXCR4 in normal aorta and AAA tissue. By transfecting the MiR206‐3p sponge to reduce the level of MiR206‐3p in AAA, the effects of the CXCL12/CXCR4 pathway on AAA progression as well as the apoptosis and phenotypic transformation of vascular smooth muscle cells (VSMCs) were studied in vivo and in vitro. Single‐cell RNA sequencing analysis, serum ELISA, and in vivo experiments indicate a pronounced activation of the CXCL12/CXCR4 axis in both AAA patients and the mouse model. Specific blocking of the CXCL12/CXCR4 axis significantly inhibited further expansion and rupture of the abdominal aorta and reduced the infiltration of inflammatory cells in the aorta and inhibited the phenotypic transformation of contractile VSMCs into a macrophage‐like state. Our findings propose that MiR206‐3p sponge represents an innovative therapeutic strategy to attenuate AAA progression and rupture risk, primarily through the suppression of the CXCL12/CXCR4 signalling pathway.

## 1 Introduction

Abdominal aortic aneurysm (AAA), a form of aortic disease, is a significant cause of mortality in adults, with an overall mortality rate exceeding 80% following rupture [1, 2, 3]. However, there is a deficiency in medications that effectively prevent aortic degeneration or decelerate the progression of AAA, particularly in cases of smaller‐diameter aneurysms or in patients with contraindications to surgery [4]. Hence, it is imperative to conduct comprehensive studies on the underlying pathologic mechanisms.

The extracellular matrix (ECM) structure of the aorta undergoes significant disruption during the progression of AAA, characterised by the rupture of the elastic membrane and excessive degradation of elastin [5]. This leads to collagen compensatory proliferation and a subsequent decrease in aortic wall elasticity. Moreover, contractile smooth muscle cells experience apoptosis and phenotypic transformation in response to various stimuli, resulting in a reduced number or density. Such alterations culminate in diminished arterial wall stiffness, ultimately causing irreversible dilatation under blood pressure [6].

Vascular smooth muscle cells (VSMCs), the most prevalent cell type in the aorta and primarily located in the media, are essential for maintaining arterial structural integrity through their contractile action and secretion of ECM components. The apoptosis and phenotypic transformation of VSMCs are believed to significantly influence AAA progression [7, 8]. Evidence suggests that promoting VSMC proliferation or inhibiting their apoptosis can lower the rupture rate of AAA [9, 10]. Yet, the phenotypic transformation of VSMCs may have a complex, bidirectional relationship with AAA progression, contingent on the type and quantity of VSMCs involved [11]. Historically, VSMCs were classified into contractile and synthetic types [12]. However, the advent of single‐cell RNA sequencing and lineage tracing technologies has facilitated the identification of a broader spectrum of VSMC types [13, 14]. Lineage tracing techniques, allow for the identification of SMCs through specific fluorescence, even after phenotypic transformation in response to inflammatory stimuli and other factors [15]. This technique is invaluable for exploring SMC phenotypic transformation, especially given the potential for the reduction or loss of SMC‐specific markers (e.g., MYH11, ACTA2, TAGLN, and CNN1) [11, 16, 17]. Recent studies indicate that VSMCs initially form clusters of intermediate cells expressing specific gene markers (e.g., KLF4 or LGALS3) before undergoing further transformation [18].

MicroRNAs (miRNAs) are endogenous regulatory RNAs, each comprising approximately 22 nucleotides. They regulate gene expression posttranscriptionally by binding directly to the 3′UTRs of target mRNAs [19]. It is estimated that miRNAs may regulate up to one‐third of human genes [20]. Recent research indicates that miR206‐3p plays a role in the progression of vascular diseases by influencing the proliferation and differentiation of skeletal and smooth muscle cells, though the underlying mechanisms remain to be elucidated [21]. Chemokines, small heparin‐binding proteins, guide inflammatory cells to sites of lesions or injuries [22]. Approximately 50 human chemokines have been identified, categorised into four main families based on their structural and functional differences; one such family is the C‐X‐C Motif Chemokine [23]. C‐X‐C Motif Chemokine Ligand 12 (CXCL12) interacts with C‐X‐C Chemokine Receptor Type 4 (CXCR4) and C‐X‐C Chemokine Receptor Type 7 (CXCR7) [24, 25]. The CXCL12/CXCR4 axis participates in numerous cellular functions, including cell proliferation, migration, and differentiation, and has been thoroughly investigated in several tumours and autoimmune diseases [26, 27, 28]. In a study involving 32 patients with AAA undergoing open surgery, CXCR4 and CXCL12 were found to be overexpressed in AAA tissue and associated with inflammation in the artery wall. Recent findings demonstrate that inhibiting the CXCL12/CXCR4 axis significantly reduces pulmonary vascular adventitial cell coverage and macrophage infiltration in pulmonary hypertension (PH) rats [29], suggesting that targeting this axis could offer a novel approach to treating aortic diseases.

Consequently, the impact of the CXCL12/CXCR4 axis on AAA development, specifically regarding diameter dilation and rupture rate, was investigated using an experimental AAA mouse model involving abdominal aortic elastase localised incubation plus oral β‐aminopropionitrile (BAPN) treatment. By transfecting the MiR206—3p sponge to reduce the level of MiR206—3p in AAA, the effects of the CXCL12/CXCR4 pathway on AAA progression as well as the apoptosis and phenotypic transformation of VSMCs were studied in vivo and in vitro.

## 2 Methods

All supporting data are available within the article and its Supporting Information. The “Expanded Materials and Methods” section is given in Supporting Information.

## 3 Results

### 3.1 Detection of CXCR12/CXCL4 Expression in VSMCs of Aortic Aneurysms

**FIGURE 1:**
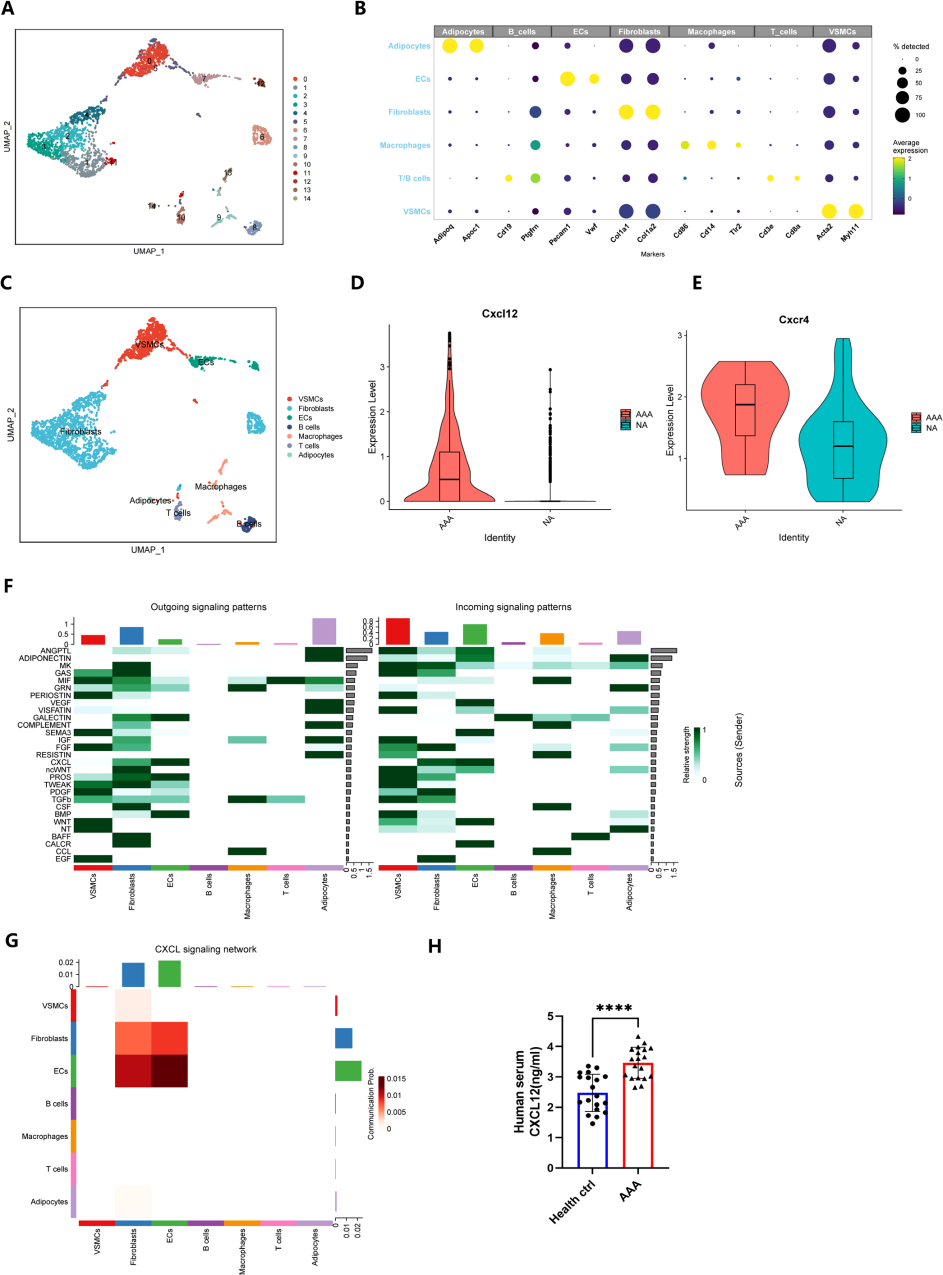
Identified CXCR4/CXCL12 expression in vascular smooth muscle cells of aortic aneurysms. (A) The UMAP projection cluster scatter diagram was noted with clusters. (B) The expression level and expression percentage of marker genes are illustrated on the dot plot. (C) The UMAP projection cluster scatter diagram was noted with cell types. (D) Expression level of Cxcl12 in healthy controls and patients with AAA. (E) Expression level of Cxcr4 in healthy controls and mice with AAA. (F) Outing and incoming signalling pathway‐mediated intercellular communication intensity was shown in the heat map. (G) CXCL signalling pathway‐mediated intercellular communication intensity was shown in hierarchy plot heat map. (H) Serum CXCL12 contents measured in patients with AAA and matched healthy individuals (*n* = 19 per group; ****p < 0.0001).

We obtained single‐cell datasets for five mouse AAA samples from the GEO database, comprising three AAA samples and two samples from normal arteries. Utilising the Seurat package in R, we categorised single cells into clusters and conducted mapping prior to single‐cell data analysis, we applied filters to remove dead or stressed cells. Subsequently, these cells were organised into 15 distinct cell populations using UMAP analysis (Figure 1A). We compared marker genes identified from previous studies with the top 50 marker genes in our dataset, ultimately identifying six cell types, including adipocytes (Adipoq, Apoc1); endothelial cells (Pecam1, Vwf); fibroblasts (Col1a1, Col1a2); macrophages (Cd86, Cd14, Tlr2); T/B cells (Cd19, Pdgfrn, Cd3e, Cd8a); and VSMCs (Acta2, Myh11) (Figure 1B,C). Our analysis revealed significantly higher expression of the CXCR4 and CXCL12 genes in AAA mice compared to normal mice, indicating enhanced activity of this inflammatory pathway (Figure 1D,E). Additionally, we visualised cell communication among different cells (Figure 1F) and observed CXCL signalling pathways between smooth muscle cells and other cells (Figure 1G). VSMCs are one of the main participants of the CXCL signalling pathway.

To bridge the gap between bioinformatics findings and clinical reality, we also measured serum CXCL12 levels in patients undergoing endoluminal repair of AAA or abdominal aortic replacement. ELISA results indicated that serum CXCL12 levels in AAA patients (*n* = 19) were significantly higher than those in healthy controls (Figure 1J), aligning with our expectations. These findings suggest the CXCL12/CXCR4 axis may play a significant role in the development of AAA.

### 3.2 Elastase‐Induced Increase of CXCL12/CXCR4 Axis Levels in AAA Mice

**FIGURE 2:**
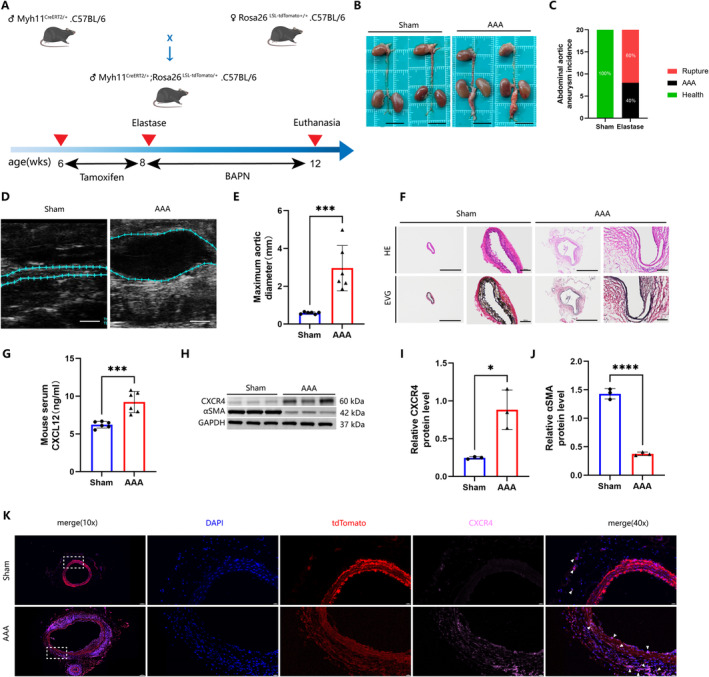
Elastase‐induced increase of CXCL12/CXCR4 axis levels in AAA mice. (A) Experimental design of AAA mouse model induced by elastase and BAPN. (B) Representative aorta photographs of sham and AAA mice (scale bar, 1 cm). (C) Abdominal aortic rupture rate in Sham and AAA groups. (D, E) Transabdominal ultrasound image of the abdominal aorta and measurements of the maximum diameter of the abdominal aorta (*n* = 6 per group; *p* < 0.001; scale bar, 1 mm). (E) Maximum abdominal aortic diameter measurement (*n* = 6 per group; *p* < 0.001). (F) Representative macroscopic images of haematoxylin and eosin (HE) and elastic‐Van Giessen (EVG) stained sections of the abdominal aorta (scale bars, left: 1000 μm, right: 100 μm). (G) Serum CXCL12 levels were detected by ELISA (*n* = 6 per group; ***p < 0.001). (H–J) Western blot analysis of CXCR4 and αSMA levels in the abdominal aorta of the healthy control and AAA mice (*n* = 3 per group; I, *p < 0.05; J, *p* < 0.0001). (K) On the basis of SMC lineage tracer tdTomato (red), immunofluorescence staining of CXCR4 (pink), and DAPI (blue) was performed and the level of CXCR4 in aorta is represented by the ratio of CXCR4 to DAPI (*n* = 6 per group; ****p < 0.0001; scale bars, left: 100 μm, right: 20 μm).

The elastase/BAPN‐induced AAA mice model, a widely utilised framework for investigating AAA, effectively generates authentic aneurysms within the abdominal aorta, contrasting with dissecting aneurysms induced by Ang II [30, 31]. Utilising an elastase/BAPN‐induced model, this study aimed to elucidate the impact of the CXCL12/CXCR4 axis on AAA progression (Figure 2A). Examination of the gross specimens revealed significant dilatation of the abdominal aorta in the model group, with aneurysms predominantly located in the infrarenal region (Figure 2B). The infrarenal abdominal aorta in certain mice experienced rapid dilation and subsequent rupture in a brief timeframe. Within 30 days of oral BAPN administration, 60% (*n* = 12) of AAA mice, compared to 0% (*n* = 0) in the sham operation group, succumbed to ruptured AAA (Figure 2C). Abdominal ultrasound assessments to measure the maximum diameter of infrarenal AAA before euthanasia showed that the maximum diameter in the AAA group was approximately five times greater than that of the sham operation group (Figure 2D,E). Haematoxylin and eosin (HE), along with elastic‐Van Gieson (EVG) staining, illustrated notable aortic diameter enlargement, increased disruption of the medial elastic membrane, and structural disarray of elastic fibres in AAA mice compared to the sham operation group (Figure 2F). To evaluate changes in the CXCL12/CXCR4 axis in AAA mice, CXCL12 levels were quantified in mouse serum via ELISA, revealing a significant elevation in AAA mice (Figure 2G), aligning with findings in AAA patients. Similarly, Western blot analysis indicated increased CXCR4 protein levels in AAA tissues (Figure 2H,I). This study utilised male Myh11^CreERT2/+^, Rosa26^tdTomato/+^ mice for the first time to conduct lineage tracing of VSMCs. Immunofluorescence for CXCR4 was employed to determine its enhanced expression in smooth muscle cells (Figure 2K). These findings suggest a significant role of the CXCL12/CXCR4 axis in the development of AAA, evidenced by its pronounced upregulation in both human and murine AAA tissues.

### 3.3 Enhancement of the CXCL12/CXCR4 Axis Is Accompanied by Increased Phenotypic Transformation and Apoptosis of VSMCs

**FIGURE 3:**
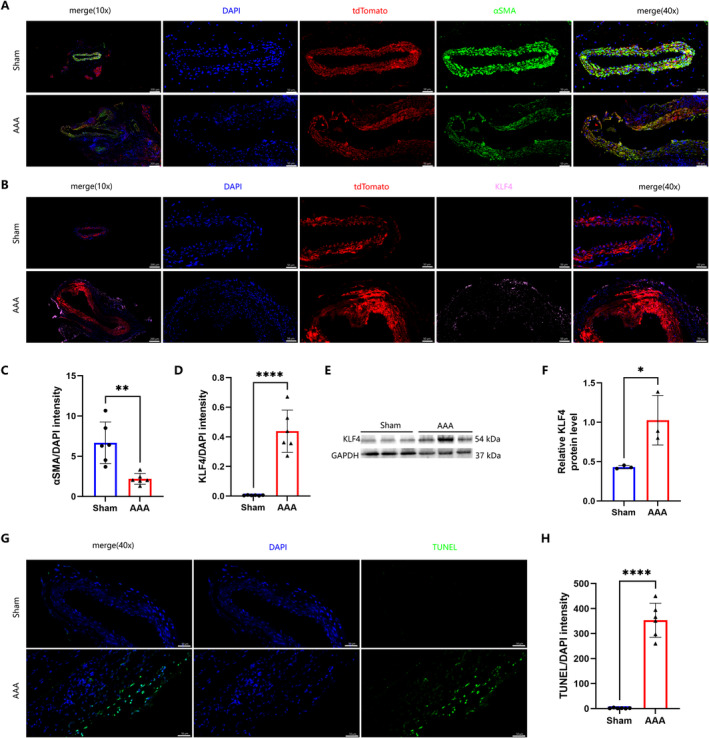
Enhancement of the CXCL12/CXCR4 axis is accompanied by increased phenotypic transformation and apoptosis of smooth muscle cells. (A, C) On the basis of SMC lineage tracer tdTomato (red), immunofluorescence staining of αSMA (green), and DAPI (blue) was performed and the level of αSMA in aorta is represented by the ratio of αSMA to DAPI (*n* = 6 per group; **p < 0.01; left: 100 μm, right: 20 μm). (B, D) On the basis of SMC lineage tracer tdTomato (red), immunofluorescence staining of KLF4 (pink) and DAPI (blue) was performed and the level of KLF4 in aorta is represented by the ratio of KLF4 to DAPI (*n* = 6 per group; ****p < 0.0001; left: 100 μm, right: 20 μm). (E, F) Representative Western blot images were used to quantitatively determine αSMA and KLF4 in mouse aortic tissue (*n* = 3 per group, *p < 0.05). (G, H) TUNEL (green) staining and quantitative determination in aorta. Nuclear antistaining with DAPI (blue; *n* = 6 per group, ****p < 0.0001; scale, 50 μm).

Contractile VSMCs undergo a phenotypic transformation in response to external injury and stimulation by circulating inflammatory factors, a process considered crucial for the progression of AAA. During this transformation, αSMA, a marker of contractile smooth muscle cells, sees a reduction in expression, whereas KLF4, indicative of a regulatory VSMC phenotype, exhibits an increase [32]. This suggests that VSMCs enter a transitional state of transformation. Utilising lineage tracing and immunofluorescence technologies enables more precise identification of transformed smooth muscle cells [15]. Lineage tracing highlighted a reduction or disappearance of αSMA expression, characteristic of over 85% of contractile VSMCs, during AAA formation (Figure 3A,C), suggesting potential misidentification of non‐αSMA expressing smooth muscle cells in the past. Furthermore, increased KLF4 expression in VSMCs (Figure 3B,D) indicates a transformation of some contractile VSMCs into other phenotypes. Western Blot analysis of abdominal aortic tissues displayed lower α‐SMA (Figure 2H,J) and higher KLF4 expression compared to normal tissues (Figure 3E,F). Concurrently, TUNEL staining confirmed heightened apoptosis in aortic smooth muscle cells (Figure 3G,H). In summary, the progression of AAA is marked by a reduction in the number or density of contractile smooth muscle cells in the aortic wall, driven by apoptosis and phenotypic transformation under the influence of the enhanced CXCL12/CXCR4 axis.

### 3.4 Inhibition of AAA Progression in Mice by CXCR4 Antagonist

**FIGURE 4:**
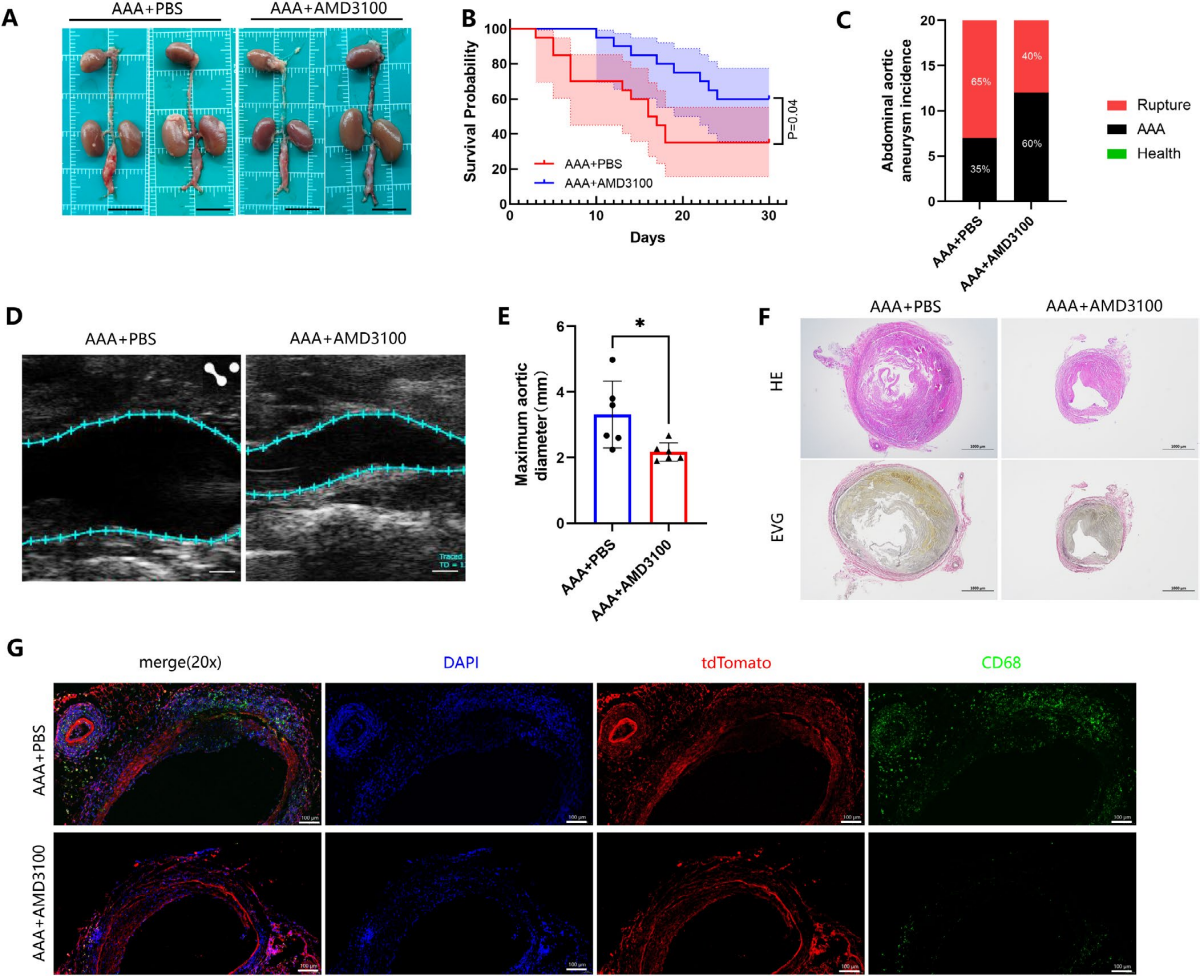
Inhibition of abdominal aortic aneurysm progression in mice by AMD 3100. (A) Representative aorta photographs of AAA mice intraperitoneally injected with PBS or AMD33100 (scale bar, 1 cm). (B) Survival probability was estimated by Kaplan–Meier method and compared by log‐rank test (*n* = 20 per group; *p* = 0.04). (C) Incidence and rupture rate of abdominal aortic aneurysm. (D, E) Transabdominal ultrasound image of the abdominal aorta and measurements of the maximum diameter of the abdominal aorta (*n* = 6 per group; *p < 0.05; scale bar, 1 mm). (F) Representative images of haematoxylin and eosin (HE) and elastic–Van Gieson (EVG) of AAA mice intraperitoneally injected with PBS or AMD33100 (scale bars, 1000 μm). (G) Representative images of mouse aorta stained with SMC lineage tracer tdTomato (red) and CD68 (*n* = 6 per group; green; scale bar, 100 μm).

Plerixafor (AMD 3100) is a specific small‐molecule inhibitor of the CXCL12/CXCR4 signalling pathway [33]. Intraperitoneal injection effectively reduced the maximum diameter of infrarenal AAA in mice (Figure 4A), resulting in a significantly lower aneurysm rupture rate in the AMD3100‐treated group (*n* = 20) compared to the control group receiving intraperitoneal injections of PBS (*n* = 20) (Figure 4B,C). Notably, the rupture rate was higher in the PBS group (65%) compared to the model group (60%), potentially due to the variations in blood pressure or body positioning during injection. Ultrasound examinations prior to euthanasia revealed that AMD3100 treatment decreased the maximum internal diameter of AAA by approximately 38% (Figure 4D,E). Histological analysis using HE and EVG staining showed that aneurysms in the PBS group had larger diameters, more disorganised elastic fibres, and increased intraluminal thrombus formation (Figure 4F). Additionally, the study observed that the inhibitor reduced CD68+ cell infiltration in the aortic wall, primarily in the adventitia and media (Figure 4G). These CD68+ cells consist of both infiltrating macrophages and macrophage‐like smooth muscle cells that carry tdTomato fluorescence. AMD3100 not only reduced the infiltration of inflammatory cells in the aortic wall, but also inhibited the phenotypic transformation of contractile VSMCs into a macrophage‐like state. These findings indicate that targeting the CXCL12/CXCR4 axis may offer an effective strategy for mitigating the progression of AAA.

### 3.5 Inhibition of AAA Progression in Mice by miR206‐3p‐Sponge Transfection

**FIGURE 5:**
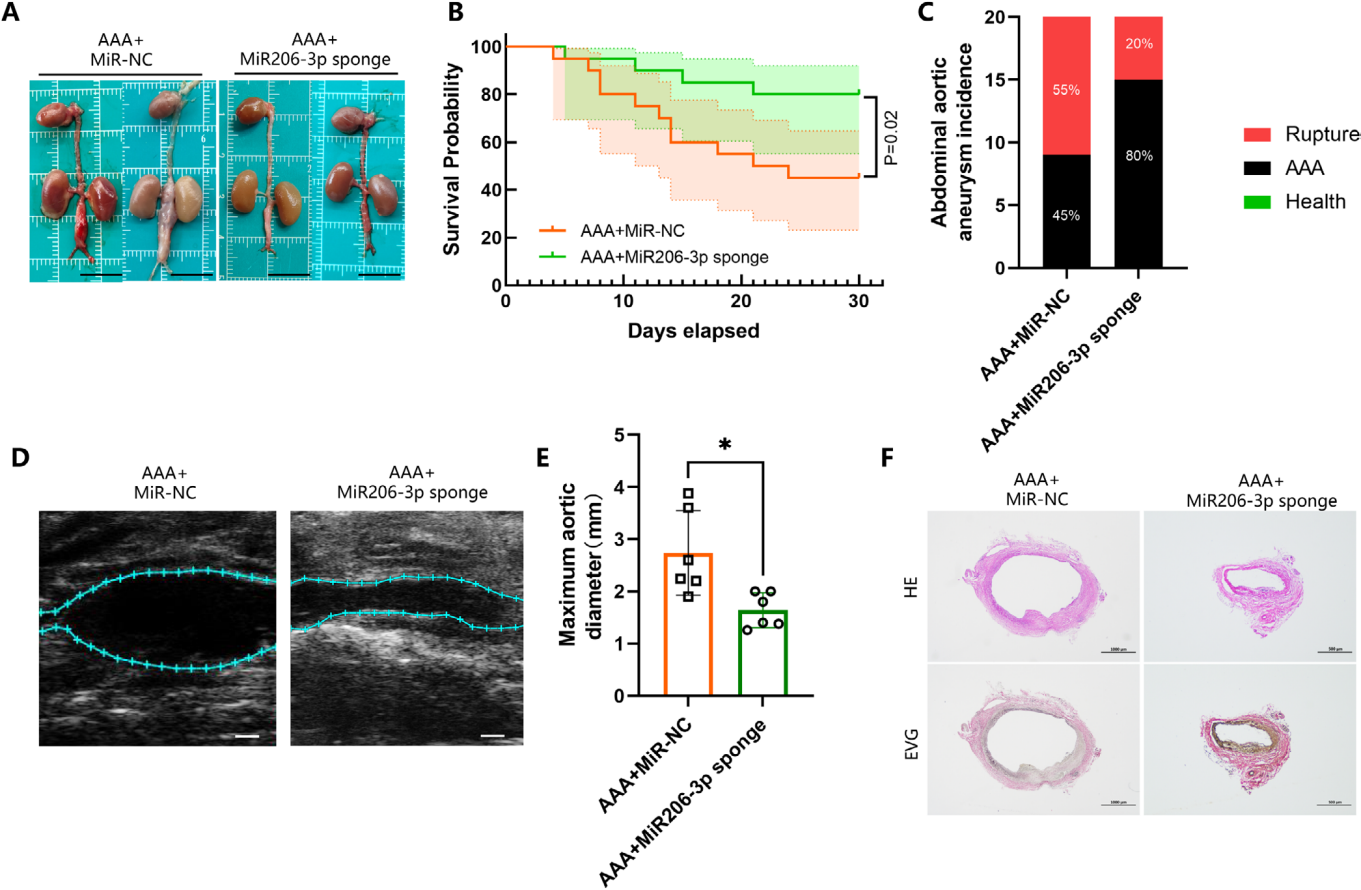
Inhibition of abdominal aortic aneurysm progression in mice by miR206‐3p‐sponge transfection. (A) Representative aorta photographs of AAA mice transfected with AAV2/9‐miR‐NC or AAV2/9‐miR206‐3p sponge (scale bar, 1 cm). (B) Survival probability was estimated by Kaplan–Meier method and compared by log‐rank test (*n* = 20 per group; *p* = 0.02). (C) Incidence and rupture rate of abdominal aortic aneurysm. (D, E) Transabdominal ultrasound image of the abdominal aorta and measurements of the maximum diameter of the abdominal aorta (*n* = 6 per group; *p < 0.05; scale bar, 1 mm). (F) Representative images of haematoxylin and eosin (HE) and elastic–Van Gieson (EVG) of AAA mice transfected with AAV2/9‐miR‐NC or AAV2/9‐miR206‐3p sponge (scale bars, 1000 μm).

MiRNA exert a crucial regulatory function in cells [34]. Notably, a single miRNA can regulate multiple mRNAs simultaneously, and conversely, multiple miRNAs can target a single mRNA, with these interactions varying significantly across different diseases [35]. In previous studies, MiR206 regulates the expression of CXCR4. Our previous research has identified a significant increase in miR206‐3p levels in AAA tissues (Figure S1). To investigate miR206‐3p's role in the development of AAA, miR206‐3p levels were reduced in these tissues using a miR206‐3p sponge gene vector delivered via adeno‐associated virus type 2/9 (AAV‐2/9). A comparison of abdominal aorta samples from the two mouse groups revealed a reduced aortic diameter in the miR206‐3p sponge transfected mice (Figure 5A). Survival analysis indicated a 35% decrease in the AAA rupture rate in the transfected group (*n* = 20) compared to the control group (*n* = 20) (Figure 5B,C). Ultrasound examinations demonstrated that the maximum internal diameter of the abdominal aorta in the transfected group was approximately 67% of that in the control group (Figure 5E). HE and EVG staining illustrated a notably smaller diameter, less disrupted elastic membrane, and more orderly elastic fibre structures in the transfected mice (Figure 5F). Collectively, these findings suggest that reducing miR206‐3p levels in AAA tissues may effectively mitigate the progression of the condition in mice.

### 3.6 Reduction of CXCR4 Expression in AAAs and Phenotypic Transformation and Apoptosis in VSMCs by miR206‐3p Sponge

**FIGURE 6:**
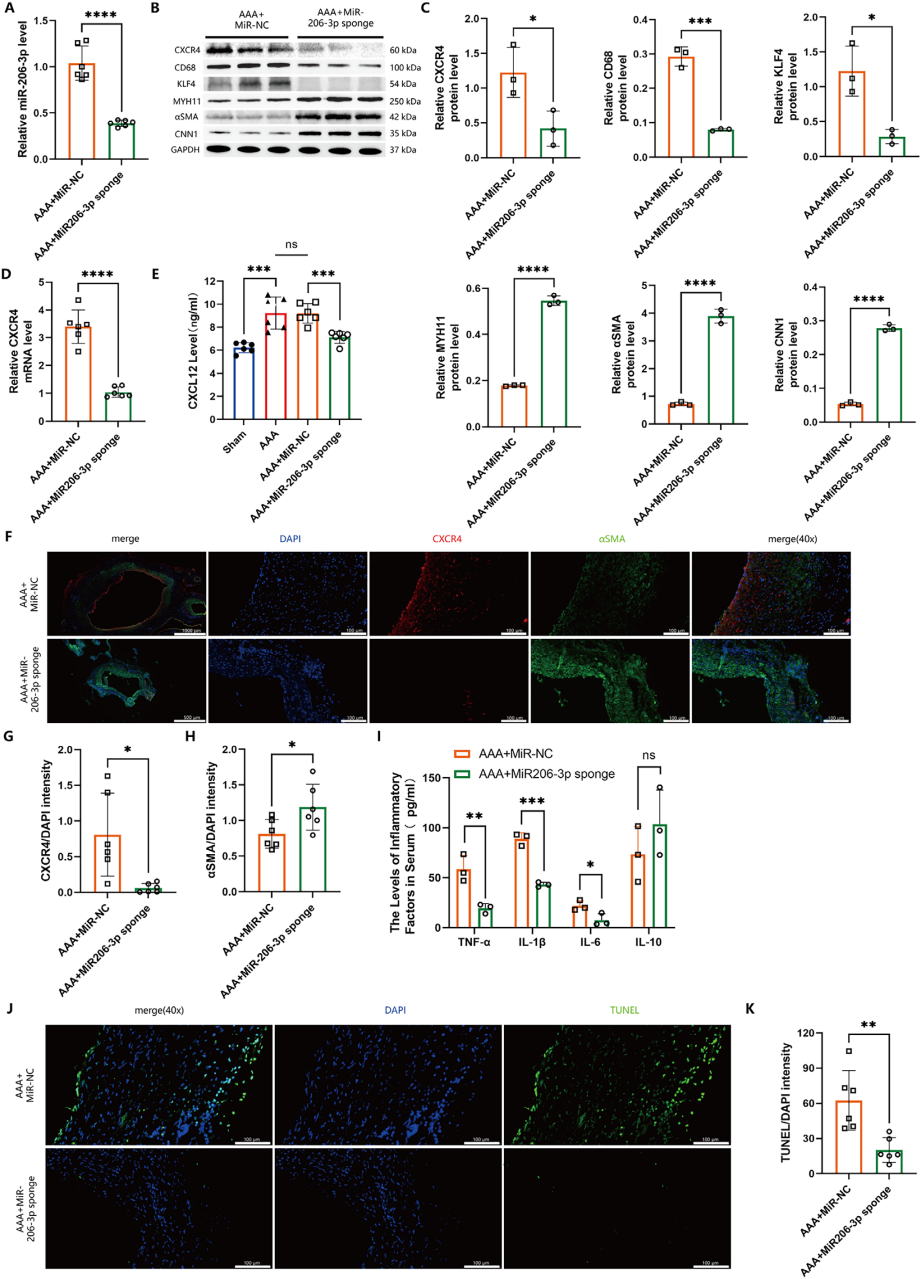
Reduction of CXCR4 expression in abdominal aortic aneurysms and phenotypic transformation and apoptosis in VSMCs by miR206‐3p sponge. (A) Relative miR206‐3p levels in abdominal aorta (*n* = 6 per group, *p* < 0.0001). (B, C) Representative Western blot images were used to quantitatively determine CXCR4 and VSMC marker proteins of different phenotypes in mouse aortic tissue (*n* = 3 per group, nonparametric Mann–Whitney *U* test, [B] *p < 0.05; [C] ****p < 0.0001). (D) Relative CXCR4 mRNA levels in aortic tissues (*n* = 6, ****p < 0.0001). (E) Serum CXCL12 levels were detected by ELISA (*n* = 6 per group; ***p < 0.001). (F–H) Immunofluorescence staining of CXCR4 (red), αSMA (green), and DAPI (blue) was performed and the level of CXCR4 and αSMA in aorta is represented by the ratio of CXCR4 and αSMA to DAPI (*n* = 6 per group; [G] *p < 0.05; [H] *p* < 0.05; left: 1000 μm, right: 100 μm). I. Serum levels of inflammatory factors were detected by ELISA (*n* = 3 per group, *p < 0.05; **p < 0.01; ***p < 0.001). (J, K) TUNEL (green) staining and quantitative determination in aorta. DAPI anti‐nuclear staining (blue; *n* = 6 per group, **p < 0.01; scale, 100 μm).

Quantitative polymerase chain reaction (qPCR) analysis revealed that miR206‐3p sponge transfection diminished miR206‐3p levels in AAA tissues by approximately 60% (Figure 6A). Subsequent analyses, including Western blot (Figure 6B,C) and immunofluorescence staining (Figure 6F,H), indicated increased expression of phenotypic transformation marker proteins (CD68 and KLF4) for VSMCs and decreased expression of marker proteins (MYH11, αSMA and CNN1) for contractile VSMCs in the tissues of the transfected group. This suggests a preservation of contractile VSMC phenotype with reduced phenotypic transformations following miR206‐3p knockdown. Furthermore, qPCR (Figure 6D), Western blot (Figure 6B,C), and immunofluorescence staining (Figure 6F,G) analyses showed that miR206‐3p sponge transfection also lowered CXCR4 expression in AAA tissues. ELISA results confirmed reduced serum levels of CXCL12 and inflammatory cytokines (IL‐6, IL‐1β, and TNF‐a) (Figure 6E,I). IL‐10 levels tended to rise in the transfected mice (*p* = 0.3041) (Figure 6I). Additionally, TUNEL staining indicated a decrease in apoptosis within the aortic tissues of the transfected group compared to the control group (Figure 6J,K). These findings suggest that lowering miR206‐3p levels in the aorta effectively inhibits phenotypic transformation and apoptosis of contractile VSMCs, potentially through the suppression of the CXCL12/CXCR4 axis in AAA.

### 3.7 Reduction of Phenotypic Transformation and Apoptosis in MOVAS by miR206‐3p‐Sponge

**FIGURE 7:**
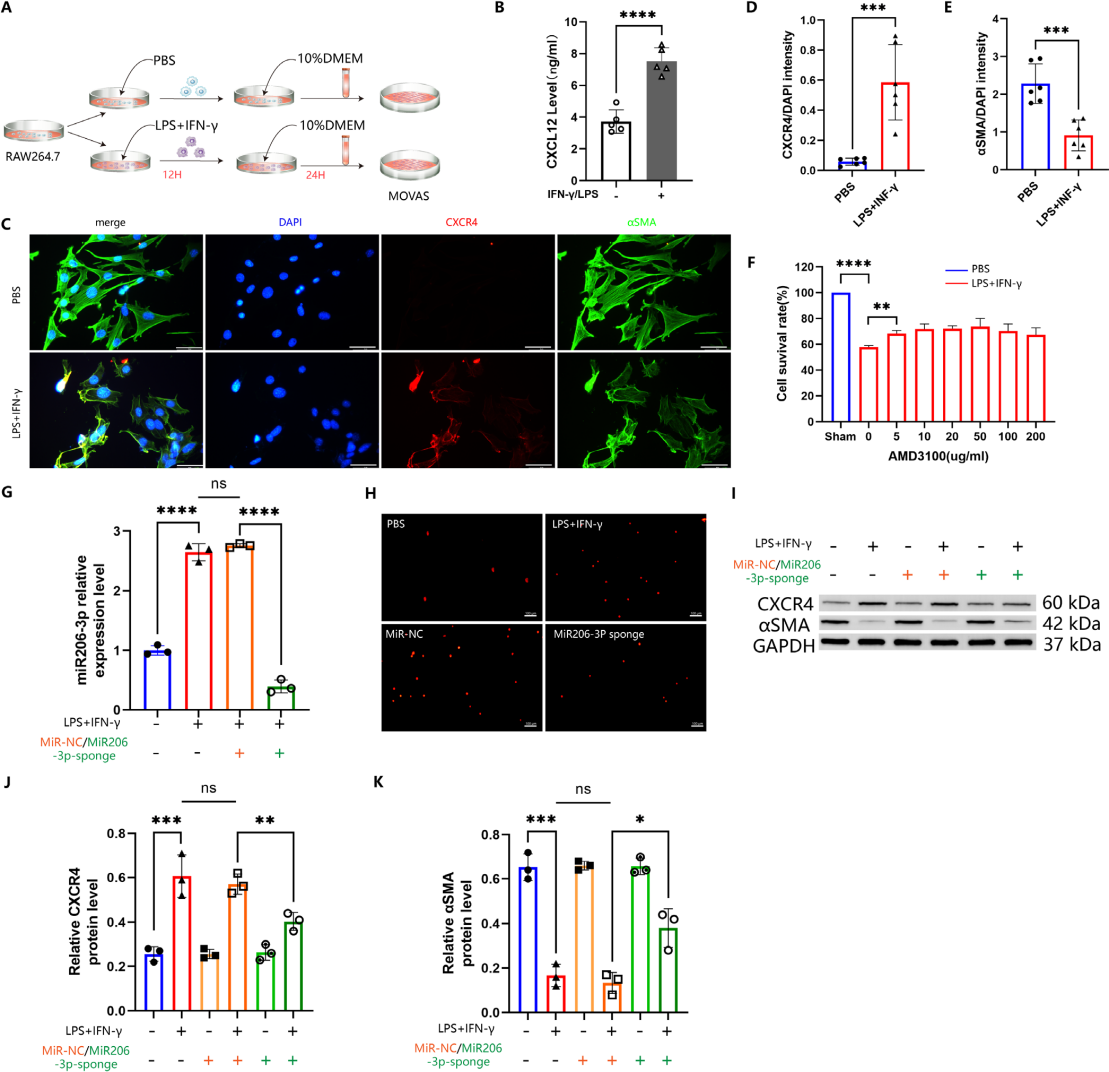
Reduction of phenotypic transformation and apoptosis in MOVAS by miR206‐3p‐sponge. (A) Illustration of the co‐culture system. LPS/IFN‐γ was used to induce RAW264.7 differentiation. The culture medium of macrophages was then used to co‐culture with MOVAS. (B) Quantification of CXCL12 content in RAW264.7 culture supernatant with or without LPS/IFN‐γ treatment (*n* = 6 per group; *p* < 0.0001). (C–E) Representative confocal images of CXCR4 (red) and αSMA (green) and filaments in VSMCs treated with or without co‐culture (scale bars, 50 μm). (F) Cell viability of MOVAS after co‐culture was detected by CCK‐8 assay. (G) Relative levels of miR206‐3p in four groups (*n* = 3 per group; ***p < 0.001; ****p < 0.0001). (H) The apoptosis of MOVAS was observed under confocal microscope (red). (I–K) Representative Western blot images were used to quantitatively determine CXCR4 and αSMA in MOVAS (*n* = 3 per group; *p < 0.05; **p < 0.01; ***p < 0.001).

The influence of CXCL12 on VSMC differentiation was further investigated through in vitro experiments. Drawing on prior research, RAW264.7 cells were stimulated with IFN‐γ/LPS for 12 h to induce CXCL12 production and secretion. Activated RAW264.7 cells exhibiting slender pseudopodia and increased iNOS expression (Figure S2). Following this, the culture medium was replaced, and the activated RAW264.7 cells were cultured for an additional 24 h. The collected culture medium was then used for the subsequent co‐culture of MOVAS cells (Figure 7A). ELISA analysis revealed significantly higher CXCL12 levels in the co‐culture medium (Figure 7B). Immunofluorescence staining of the cells showed a marked increase in CXCR4 expression and a decrease in αSMA expression, after 24 h of co‐culture (Figure 7C–E). CCK‐8 assays indicated a reduction in MOVAS viability post co‐culture, which was partially reversed by AMD3100 treatment (Figure 7F). To assess the impact of reduced miR206‐3p levels on smooth muscle cells within the AAA microenvironment, miR206‐3p sponge was introduced via lentiviral transfection (Figure 7G). The co‐culture heightened smooth muscle cell apoptosis, with a significantly reduced apoptosis rate observed in the transfected MOVAS group (Figure 7H). Furthermore, Western blot analysis demonstrated elevated αSMA protein levels and decreased CXCR4 protein levels in MOVAS cells from the transfected group compared to the control group (Figure 7I–K). These findings indicate that the in vitro results corroborate the in vivo observations.

## 4 Discussion

Our study validates that inhibiting the CXCL12/CXCR4 axis can mitigate AAA progression and lower rupture risk, as evidenced by both in vivo and in vitro experiments, highlighting the therapeutic potential of CXCR4 targeting for AAA management. Additionally, we demonstrate that miR206‐3p deficiency plays a pivotal role in hindering AAA progression, potentially through CXCL12/CXCR4 axis inhibition, underscoring the therapeutic promise of miR206‐3p sponge in AAA treatment.

Chemokines, structurally akin small molecule secreted proteins with molecular weights around 8–10 kDa, facilitate cell migration and activation by binding to their complementary G protein‐coupled cell surface receptors. These molecules are crucial for therapeutic interventions in human diseases, as they modulate inflammatory responses in pathological conditions [36]. CXCL12's interaction with CXCR4 initiates downstream signalling pathways involved in various cellular processes, including proliferation, migration, and differentiation [37]. Originally identified as a co‐receptor for HIV‐1 in CD4+ T cell infection [38], CXCR4 expression has been detected in numerous cancer cells [39], often overexpressed at tumour sites alongside elevated stromal tissue CXCL12 levels. Recent research by Bordenave et al. and Michineau et al. has shown that blocking the CXCL12/CXCR4 axis significantly reduces pulmonary vascular epicardial cell coverage and macrophage infiltration in PH rats [29], Michineau et al. demonstrated through a GFP+/− bone marrow transplantation experiment that overexpression of CXCL12 in AAA was linked to the recruitment of bone marrow‐derived macrophages. Furthermore, real‐time qPCR analysis indicated elevated levels of both CXCL12 and CXCR4 mRNA in the AAA wall of human and CaCl2‐induced mice [40]. Given the critical role of CXCR4 in the signalling network associated with arterial disease, we are keenly interested in developing therapeutic strategies for this target.

AMD3100 was initially identified as a contaminant in a commercial cyclam sample [41], which was subsequently discovered to specifically inhibit CXCR4 signalling [42]. As a result, AMD3100 has become increasingly utilised in basic research targeting the CXCL12/CXCR4 pathway disorders, including as a specific inhibitor of CXCR4. Chronic autoimmune diseases such as rheumatoid arthritis (RA) and systemic lupus erythematosus involve multiple sites with high levels of inflammatory cells and cytokines. AMD3100 or its derivatives have been shown to inhibit the migration of CXCR4+ cells into the synovial fluid of RA patients, reduce neovascularisation, and lessen the severity of lupus nephritis in an arthritic mouse model [39]. Bordenave et al. similarly found that AMD3100 significantly reduced macrophage infiltration in the pulmonary arteries [29]. Our research confirms that AMD3100 curtails the progression of AAA and lowers their rupture risk while diminishing CD68+ cell infiltration in the aortic wall, aligning with earlier findings. Traditionally, smooth muscle cells were categorised as either contractile or synthetic. However, with advances in single‐cell sequencing and lineage tracing technologies, an increasing variety of smooth muscle cell types has been identified, including macrophage‐like, mesenchymal‐like, fibroblast‐like, adipocyte‐like, T‐cell‐like, and osteochondrogenic cell‐like states [43]. The phenotypic transformation of VSMCs is characterised by a decline in the expression of contractile‐specific markers (e.g., MYH11, αSMA, CNN1) and an increase in markers associated with other cell types (e.g., CD68, CD34, FABP4, CD3D). These cells exhibit reduced contractile function and acquire capabilities, such as phagocytosis and synthetic secretion. Recent studies have elucidated that VSMCs initially form clusters of intermediate cells (i.e., modulated VSMCs) expressing genetic markers (e.g., KLF4 and Lgals3), which then transform into other types. This process was corroborated in our study, where KLF4 protein levels were markedly elevated in AAA tissues, but contractile phenotypic marker αSMA levels were decreased. AMD3100 not only reduced the infiltration of inflammatory cells in the aortic wall, but also inhibited the phenotypic transformation of contractile VSMCs into a macrophage‐like state.

Located on chromosome 6p12.2, the gene encoding miR206 exhibits a highly conserved genomic sequence as a vertebrate‐specific miRNA [44]. MiR206‐3p has been extensively demonstrated in previous studies to inhibit the proliferation of various cancers [19, 45]. Additionally, research by HK Kim et al. determined that overexpression of miR206 enhances the differentiation of C2C12 myoblasts. Subsequent findings indicated that decreasing miR206 levels encourages smooth muscle cell proliferation. Our findings further reveal that reducing intracellular miR206‐3p levels through the transfection of MiR206‐3p‐sponge improves VSMC viability and lowers apoptosis rates in VSMCs in both in vivo and in vitro settings, contributing to the preservation of arterial wall elasticity and mitigating the risk of expansion and rupture of AAA. In Wright's study, miR206 was thought to inhibit CXCL12/CXCR4 signalling and prevent neutrophil recruitment to granulomas [46]. However, our results show that when miR206‐3p levels in VSMCs are reduced, CXCL12/CXCR4 axis activity is significantly inhibited. The infiltration of CD68+ cells (considered macrophages or macrophage state VSMCs) in the aorta is reduced, as is the level of circulating inflammatory factors. Although previous studies have suggested that there is a regulatory relationship between miR206 and CXCR4, we believe that the effect of MIR206‐3p sponge may be an indirect beneficial effect in AAA, which may be due to the maintenance of contractional VSMC phenotype, reduction of phenotypic transformation, and reduction of apoptosis. At the same time, we also observed that the miR206‐3p sponge group had lighter ECM destruction, and in this relatively physiological cell microenvironment, the CXCL12/CXCR4 axis activity was significantly inhibited. This may be the reason for our observations, which we report in this paper and look forward to receiving more attention and discussion. In the future, we will also further design experiments to observe the intervention at different stages of AAA formation and further explore the mechanism behind this phenomenon by using gene editing and other technologies.

AMD3100 exerts a systemic effect. Current literature indicates that, besides typical adverse effects, it perturbs the haematopoietic system, inducing bone marrow mobilisation [47]. In contrast, miRNAs, like miR206‐3p, can finely regulate disease‐related signalling pathways by modulating gene expression, potentially impeding disease progression more effectively. To enhance miR206‐3p's therapeutic potential for AAA, specific strategies are adopted. AAV 2/9, known for good transduction efficiency and biocompatibility, is used as a carrier. Coupled with VSMC‐specific promoters that activate only in AAA‐relevant diseased VSMCs, this enables targeted delivery and tissue‐specific expression of miR206‐3p. Consequently, it improves miR206‐3p's efficacy and safety in AAA treatment, presenting a promising prospect for clinical applications and translational research.

However, an in‐depth analysis of the underlying molecular mechanisms was not conducted in this study. Despite the emerging use of miRNAs in gene therapy for certain genetic disorders, there remains a considerable journey ahead. Our study contributes to understanding the pathogenesis of AAA and suggests new therapeutic strategies for the condition. It is worth to further study and explore the possibility of clinical transformation.

## Author Contributions

**Xuezhen Xuan:** conceptualization (equal), data curation (equal), investigation (equal), methodology (equal), validation (equal), visualization (equal), writing – original draft (equal). **Yaling Li:** conceptualization (equal), investigation (equal), methodology (equal), validation (equal), writing – original draft (equal). **Genmao Cao:** conceptualization (equal), investigation (equal), validation (equal). **Jie Hu:** formal analysis (equal), project administration (equal), software (equal). **Sheng Yan:** software (equal), visualization (equal). **Haijiang Jin:** funding acquisition (equal), project administration (equal), visualization (equal). **Maolin Qiao:** investigation (equal), software (equal). **Ruijing Zhang:** project administration (equal), resources (equal), supervision (equal), writing – review and editing (equal). **Honglin Dong:** resources (equal), supervision (equal), writing – review and editing (equal).

## Conflicts of Interest

The authors declare no conflicts of interest.

## Supporting information


Appendix S1.

## Data Availability

The data that supports the findings of this study are available in the Supporting Information of this article.
