# Supplementary material for: Inhibition of Abdominal Aortic Aneurysm Progression Through the CXCL12/CXCR4 Axis via MiR206‐3p Sponge
Source: J Cell Mol Med. 2025 Jan 8;29(1):e70328. doi: 10.1111/jcmm.70328 (PMC11710933; doi:10.1111/jcmm.70328)
Supplement: Supplementary file 1 — Appendix S1. [file JCMM-29-e70328-s001.zip › jcmm70328-sup-0002-TableS1.docx]

**Supplementary Material**

Supplementary Table. Patient Characteristics

| Characteristics | AAA | Healthy ctrl |
| --- | --- | --- |
|  | n=19 | n=19 |
| Age（y） | 68.8±10.1 | 68.8±9.6 |
| Male | 4(21.1%) | 4(21.1%) |
| Coronary heart disease | 0(0.0%) | 0(0.0%) |
| Hyperlipidemia | 0(0.0%) | 0(0.0%) |
| Diabetes mellitus | 1(5.3%) | 1(5.3%) |
| Hypertension | 8(42.1%) | 2(10.5%) |
| Smoking | 9(47.4%) | 4(21.1%) |
| Maximum abdominal aortic diameter (cm) | 5.28±1.0 | NA |

Data are expressed as a number (percent) or as the mean ± standard deviation. AAA: patients diagnosed with abdominal aortic aneurysm, serum is used. Healthy ctrl: age-matched healthy people committed to physical examination, serum is used.

Supplementary Figure 1


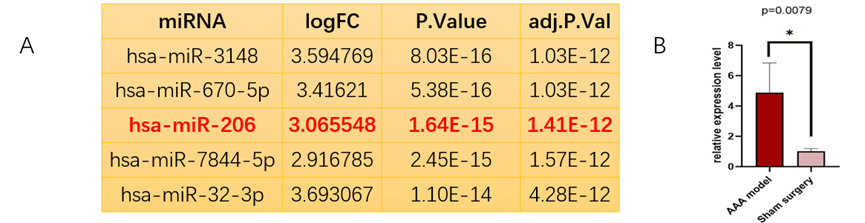


A. Differential analysis of aortic aneurysm miRNA sequencing dataset GSE110527 was performed using Limma package, and the top 5 highly expressed mirnas were calculated. B. Relative levels of miR206-3p in abdominal aorta were detected by RT-PCR (n=3, P=0.0079).

Supplementary Figure 2


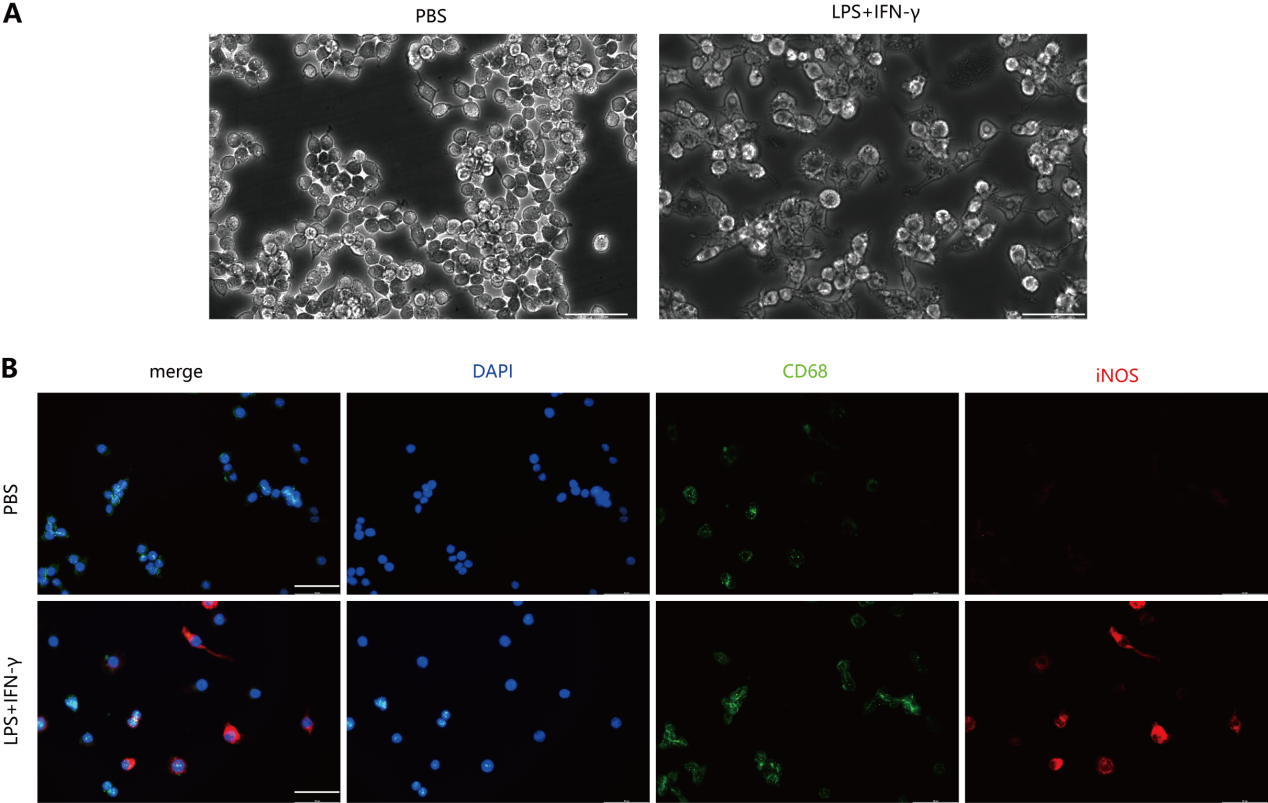


1. Morphological changes of RAW264.7 cells after induction by LPS/IFN-γ under light microscope(scale bars, 50 μm). **B**. Representative confocal images of iNOS (red) and CD68 (green) in RAW264.7 cells treated with or without LPS/IFN-γ (scale bars, 50 μm).

**Methods**

**Data Source and Data Pre-processing**

Single-cell sequencing data for three AAA mouse aortic aneurysm samples and two mouse normal aorta samples were obtained from GSE186865. Aortic aneurysm samples and normal aorta samples were merged to create two Seurat objects, respectively, by using Seurat R package (version 3.1.2). Subsequently, data integration was performed between the aortic aneurysm Seurat object and the normal aorta Seurat object by identifying anchors between the two datasets. Cells with less than 200 genes or more than a 10% mitochondria content and genes with less than 10 cells were removed. The count matrix was normalized and scaled by “NormalizeData” function and “ScaleData” function in Seurat, respectively.

**General Cell Type Identification Identification**

Top 2000 highly variable genes (HVGs) were calculated through “FindVariableFeatures” function in Seurat, and top 20 principal components were calculated by “RunPCA” function according to the top 2000 HVGs. The UMAP algorithm with a solution of 0.6 was used for clustering and visualization of all cell clusters. The automatic cell annotation algorithm “SingleR” annotated all cell clusters by using databases “HumanPrimaryCellAtlasData” and “BlueprintEncodeData” as the reference. Background knowledge pertaining to the cellular components of normal aortas and aortic aneurysms was used to assist cell annotation. VSMCs identified by the aforementioned steps were separately extracted, and HVGs and the top 20 principal components were recalculated for re-clustering.

**General Intercellular Communication Analysis**

R package “CellChat” (version 1.1.3) is the latest algorithm that infers intercellular communication from gene expression levels from single-cell transcriptome data. All celltypes were extracted for intercellular communication analysis. The gene expression profile of extracted cells was input for identifying differentially overexpressed ligands and receptors of each cell population. “CellChat” first calculated a probability value of each ligand–receptor interaction, and then the communication probability of each signaling pathway was calculated by summarizing the probability of its subordinate ligand–receptor pairs. The communication probability refers to communication strength. Heatmap was plotted for picturing communication probabilities between all cell pairs.

**Human Serum Collection and Ethics Statement**

All patients with abdominal aortic aneurysms are diagnosed by abdominal vascular ultrasound or CTA by experienced vascular surgeons and imaging physicians. This study was approved by the Ethics Committee of the Second Affiliated Hospital of Shanxi Medical University (approval document No. (2023) YX No. (271)) and conducted in accordance with the Declaration of Helsinki. All patients received written informed consent. Serum was collected from AAA patients who were expected to undergo endovascular isolation for abdominal aortic aneurysm. Control serum was collected from age-matched healthy individuals who had no aneurysm, dissection, coarctation, or previous aortic repair. The characteristics of all patients with abdominal aortic aneurysms who participated in this project and the matched healthy population were recorded in (Supplementary Table).

**Animals and mouse model**

Our research program was approved by the Animal Ethics Committee of the Second Hospital of Shanxi Medical University (approval number DW2023048). To construct pedigree tracer mouse strains, Myh11promoter-CreERT2 sequence was inserted into the Y chromosome of C57BL/6 mice, and obtained Myh11^CreERT2/+^ mice after screening. Then Myh11^CreERT2/+^ mice and Rosa26^LSL-tdtomato+/+^ mice (purchased from Shanghai Nannan Model Biotechnology Co., LTD.) were hybridized to obtain male Myh11^CreERT2/+^; Rosa26^tdTomato/+^ mice. 6 weeks old male Myh11^CreERT2/+^; Rosa26^tdTomato/+^ mice were used for lineage tracing of vascular smooth muscle cells. Before the experiment, the mice were continuously intrabitoneally injected with Tamoxifen (50mg/kg/day) for 10 days to induce expression of tdTomato protein. We stratified mice according to body weight and randomly divided them into sham group, AAA group, miR-NC group and miR206-3p sponge transfection group. Each mouse was anesthetized by inhalation of isoflurane and a midline incision was made through the abdominal skin and muscle. The abdominal aorta and surrounding tissue were carefully separated under a light microscope. Subsequently, gelatin sponge impregnated with elastase solution (50mg/ml) was placed close to the lower renal segment of abdominal aorta for 15min. The sham group used gelatin sponges impregnated with 0.9% saline. After the sponge was removed, the abdominal cavity was lavished three times with 0.9% saline. After resetting the abdominal organs, the muscles and skin were sutured with 6-0 nylon thread. The mice were fed 0.2% fumarate 3-amino-propionitrile (BAPN) for 30 days after surgery. AMD3100 (Abcam,ab120718), a specific inhibitor of CXCR4, was dissolved in PBS and injected intraperitoneally for 30 days(5mg/kg/day). Sham animals received vehicle treatment. AAV2/9 containing a plasmid carrier of miR-NC or miR206-3p sponge was injected through the tail vein 30min after the completion of elastase incubation. The titers and volumes of the two groups of AAVs were AAV2/9-ZsGreen NC (titer: 1.2×1012 vp/ml, 70 μl) and AAV2/9-CMV-mmu-miR-206-3P-SP onge ZsGreen (titer: 1.4×1012 vp/ml, 60 μl) to construct transfection model. Objective Gene sequence information: mmu-miR-206-3p：

UGGAAUGUAAGGAAGUGUGUGG，mmu-miR-206-3p-sponge：

CCACACACTTAAGACATTCCAtatacCCACACACTTAAGACATTCCAacatcCCACACACTTAAGACATTCCAtcttcaCCACACACTTAAGACATTCCA

**Cell culture, treatment and transfection**

Mouse aortic smooth muscle cells (MOVAS) and mouse mononuclear macrophage leukemia cells (RAW264.7) were purchased from Jiangsu Kaiji Biotechnology Co., LTD. (article No. KG612) and Suzhou Haixing Biotechnology Co., LTD. (TCM-C766), respectively. We used DMEM medium containing 1% penicillin/streptomycin and 10% fetal bovine serum (FBS) for cell culture and placed the medium in a 37°C incubator containing 5% CO2. To construct a stable transfection cell line, we infected MOVAS with a miR206-3p sponge expressing lentivirus or lentiviral vector (MOI=100) in complete culture medium for 72 hours. Transfection efficiency was calculated after 48 hours of culture. In addition, 2.0ug/ml puromycin (China, article No. HB-PU-500) was used to screen lentivirus stable strains. After obtaining the stable strains, the cells were amplified by passage. In order to provide AAA microenvironment, we added induced RAW264.7 (2.5ng/ml IFN-γ+200ng/ml LPS) culture medium to MOVAS and continued to culture for 24 hours.

**Animal ultrasound**

The maximum abdominal aortic diameter was assessed using the Vevo 3100 platform (FUJIFILM VisualSonics) and the MX550D transducer (FUJIFILM VisualSonics) before mouse AAA modeling and before execution. Mice were anesthetized with isoflurane (3% isoflurane for induction and 2% isoflurane for maintenance) and placed on a heated plate at 37 ℃. We selected the most severely dilated area of the subrenal abdominal aorta and used longitudinal B-mode ultrasound to obtain maximum intra-aortic diameter during systole. In all abdominal ultrasound experiments, the mice were tested in a random order.

**microRNA analysis by real-time PCR**

The aorta tissues of mice were rapidly isolated, and total microRNA (Mei5 Biotechnology, Cat.MF044-01) was extracted by centrifugal-adsorbed silica matrix membrane separation after liquid nitrogen grinding. The HyperScriptTM III miRNA 1st Strand cDNA Synthesis Kit(by stem-loop)(NovaBio # R601) reverse transcriptase was then used to reverse transcribe the cDNA. The expression of miR206-3p was detected by RT-PCR (NovaBio # Q206). The expression level of miRNA was normalized to U6 level and calculated by △△CT method. All reagents are used in accordance with the manufacturer's instructions. The amplification reaction procedure consists of predenaturation at 95℃, 30s, followed by 40 cycles at 95 ℃, 10s, and 60℃, 30s. The primers for miR206-3p (RT primer: (Stem-loop RT Primer: 5’GTCGTATCCAGTGCAGGGTCCGAGGTATTCGCACTGGATACGACCCACAC3’; qPCR forward primer:5’GCGCGTGGAATGTAAGGAAGT3’; qPCR reverse primer:5’AGTGCAGGGTCCGAGGTATT3’), U6 (RT primer: (Stem-loop RT Primer:5’GTCGTATCCAGTGCAGGGTCCGAGGTATTCGCACTGGATACGACA

AAATATGG3’; qPCR forward primer:5’GCTCGCTTCGGCAGCACATATAC3’; qPCR reverse primer:5’AGTGCAGGGTCCGAGGTATT3’)were designed by EnzyArtisan (Shanghai, China). The RNA isolation of MOVAS and real-time PCR were the same as before.

**Western Blot**

Protein was extracted from MOVAS and abdominal aorta tissues using RIPA lysis buffer (Thermo Scientific, Cat#89901). To put it simply, the rat abdominal aortic segment tissue or MOVAS was rapidly divided after the experiment was terminated, treated with ultrasonic crusher (manufacturer, model), cracked with RIPA cracking solution, and then obtained cell or histopin solution after centrifugation with supernatant and boiling water bath denaturation. An equal amount of protein extract (20~30μg/lane) was analyzed by Western blot analysis on 10%SDS-PAGE (Servicebio, Cat#G2003-50T, Wuhan, China) and transferred to PVDF membrane. After being blocked with 5% skim milk powder at room temperature for 2h, the primary antibody CXCR4(Abcam,ab181020,dilution 1:1,000), a-SMA (Abcam, ab124964, dilution 1:20,000) , KLF4(Abcam, ab214666, dilution 1:1,000), anti-CD68 (Abcam, ab283654, dilution 1:5,000), CNN1 (Abcam, ab46794, dilution 1:5,000) and anti-MYH11 (Abcam, ab124679, dilution 1:30,000) were incubated at 4℃ overnight, and the secondary antibody was incubated at room temperature for 1h. The image was obtained by the ChemiDoc XRS+ system (Bio-Rad). In this study, the ECL protocol was used, GAPDH was used as endogenous reference, and goat anti-mouse or rabbit anti-mouse IgG was used to detect antigen antibody complex.

**Cytotoxicity** **assay**

Cell viability was measured by CCK-8 Cytotoxicity and viability assay kit (GLPBIO,GK10001). Simply put, MOVAS suspension was inoculated into a 96-well plate at a density of 4*10^3^ cells/well and cultured in 100ul medium for 24h (37℃, 5% CO2). After that, the activated macrophage supernatant and AMD3100 solution of different concentrations were added to the plate. After the 96-well plates were incubated in the incubator for 24h, they were washed 3 times with PBS, and the culture medium was replaced with fresh medium containing 10% CCK-8 proliferation kit. After two hours of incubation, the absorbance at 450 nm was measured using an enzymograph. Cell viability was measured and normalized with control group. Finally, Prime 9 was used for statistical analysis of cell viability under each AMD3100 concentration.

**TUNEL assay**

Cells or abdominal aorta segments were fixed with 4% paraformaldehyde and paraffin embedded, histological sections (3um) were performed. After the sections were dewaxed, 20μg/ml protease K was added and incubated in 37℃ incubator for 30min. After that, the TUNEL test solution was added to 37°C incubator and incubated for 60min in the dark, and then washed with PBS for 3 times. Droplet nuclei were backstained with DAPI. After dehydration and transparency, anti-fluorescence quenching sealing tablets are added. Red fluorescence was observed using confocal microscopy. Images were obtained by Leica Application Suite X Software (Leica, DMIL LED Inverted Microscope).

**ELISA**

After the experiment, the mice were euthanized and their blood was centrifuged to obtain serum. Human stromal cell derived derived factor 1 (SDF-1) ELISA kit (MEIMIAN, Cat#MM-2124H1, Jiangsu, China) was used in strict accordance with the instructions to detect the serum CXCL12 levels of the patients. For the detection of CXCL12, IL-6, TNFα, IL-1β, and IL-10 levels in mouse serum and cell supernatants, the following ELISA kits were employed: the mouse stromal cell-derived factor 1 (SDF-1) ELISA (MEIMIAN, Cat# MM-44155M1, Jiangsu, China), Mouse IL-6 Uncoated ELISA (invitrogen, Cat# 88-7064), Mouse TNF alpha Uncoated ELISA (invitrogen, Cat# 88-7324), Mouse IL-1 beta Uncoated ELISA (invitrogen, Cat# 88-7013), and Mouse IL-10 Uncoated ELISA (invitrogen, Cat# 88-7105).

**Immunofluorescence Staining**

After euthanized mice, abdominal aorta tissue was removed and OCT embedding was performed. The tissues were then frozen and fixed with 4% paraformaldehyde for 30min. The sections were repaired with EDTA antigen repair buffer (PH6.0) and blocked with 5% BSA for 30min. The slices were then incubated overnight with anti-CXCR4 (Abcam, ab181020,dilution 1:500), anti-αSMA (Abcam, ab124964 ,dilution 1:500 and ab7817,1 µg/ml), anti-KLF4 (Abcam, ab214666,dilution 1:1,000) and anti-CD68 (Abcam, ab283654,dilution 1:50) antibodies at 4°C. Secondary antibodies (Abcam, ab150117 and ab150080,dilution 1:500) were incubated in the dark at room temperature for 50min. The nucleus is marked blue by DAPI. The immunofluorescence effect was observed immediately after sealing. When cell immunofluorescence staining was performed, MOVAS cells located on the cell crawl were first immobilized with 4% paraformaldehyde, followed by penetration with 0.3% Triton X-100 for 3min and blocked with 1:20 diluted goat serum. Anti-CXCR4 (Abcam, ab181020,dilution 1:500) and anti-αSMA (Abcam, ab124964,dilution 1:500 and ab7817,1 µg/ml) antibodies were incubated overnight at 4℃. Secondary antibodies (Abcam, ab150117,dilution 1:500 and ab150080,dilution 1:500) were incubated in the dark at room temperature for 2h. Nuclear staining with DAPI. The immunofluorescence effect was observed immediately after sealing. Immunofluorescence images were obtained by Leica Application Suite X software (Leica, DMIL LED inverted microscope).

**Hematoxylin &** **Eosin Staining and Elastic Fiber Staining**

After the end of the experiment, the abdominal aortic segment of the rats was quickly removed, and the maximum diameter segment of the abdominal aortic aneurysm was cut off, fixed in 4% paraformaldehyde and paraffin embedded for histological section (3um). hematoxylin & eosin staining (HE), elastic-Van Gieson (EVG) staining was conducted in strict accordance with the requirements of reagent suppliers.

**Statistical Analysis**

The results were expressed as mean ±SD, and statistical analysis was performed using GraphPad Prism 9.0. Results are expressed as mean ± standard deviation. The Student t test was used for statistical comparison between the two groups. One-way analysis of variance was performed for three or more groups. P≤0.05 was considered statistically significant.
