# Supplementary material for: Inhibition of Abdominal Aortic Aneurysm Progression Through the CXCL12/CXCR4 Axis via MiR206‐3p Sponge
Source: J Cell Mol Med. 2025 Jan 8;29(1):e70328. doi: 10.1111/jcmm.70328 (PMC11710933; doi:10.1111/jcmm.70328)
Supplement: Supplementary file 1 — Appendix S1. [file JCMM-29-e70328-s001.zip › jcmm70328-sup-0001-FiguresS1.docx]

Supplementary Figure 1


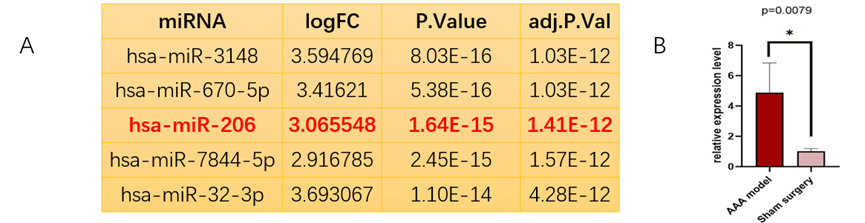


A. Differential analysis of aortic aneurysm miRNA sequencing dataset GSE110527 was performed using Limma package, and the top 5 highly expressed mirnas were calculated. B. Relative levels of miR206-3p in abdominal aorta were detected by RT-PCR (n=3, P=0.0079).

Supplementary Figure 2


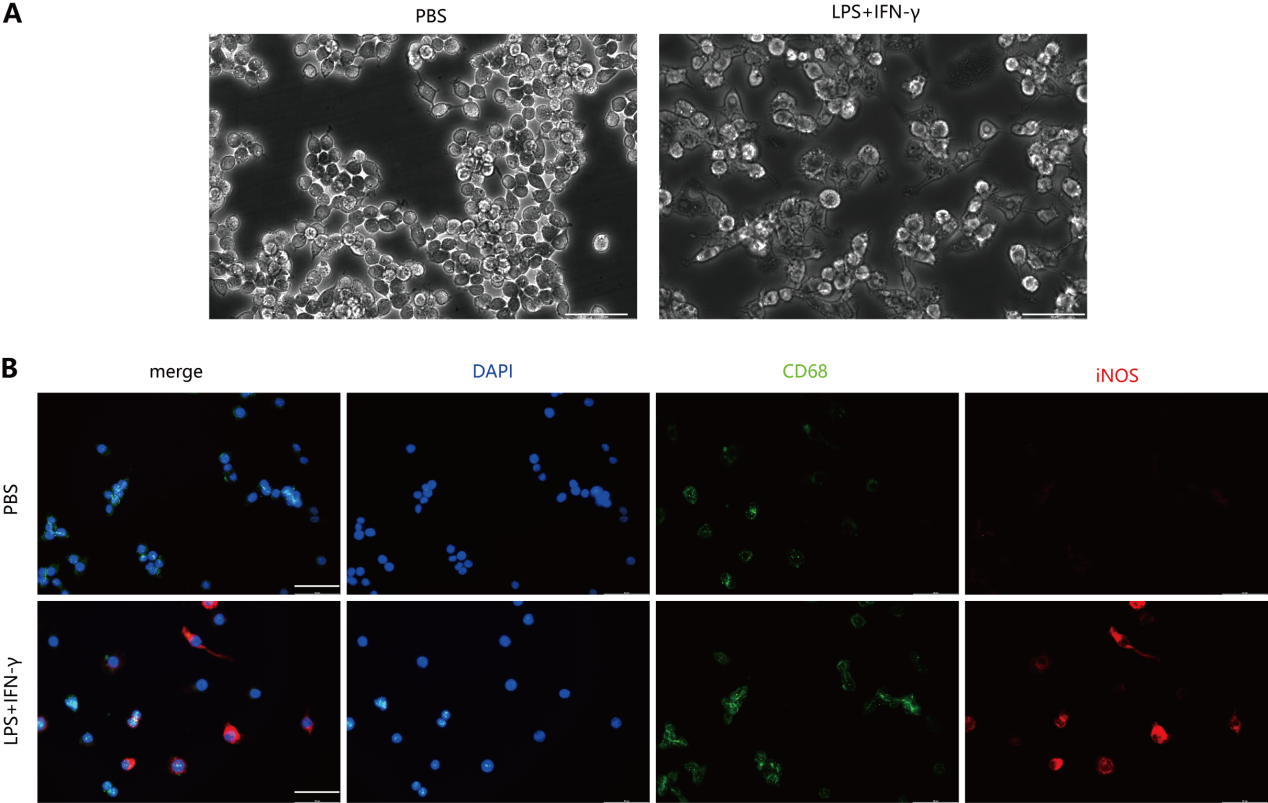


1. Morphological changes of RAW264.7 cells after induction by LPS/IFN-γ under light microscope(scale bars, 50 μm). **B**. Representative confocal images of iNOS (red) and CD68 (green) in RAW264.7 cells treated with or without LPS/IFN-γ (scale bars, 50 μm).
